# Informal Caregivers’ Health Literacy and Nursing Practices Perceived as Supportive of Health Literacy in Primary Care: A Sequential Mixed-Methods Study

**DOI:** 10.3390/healthcare14182987

**Published:** 2026-09-12

**Authors:** Carina Domingues Antunes, Alcinda Reis, Celeste Godinho, Ana Cristina Spinola Madeira

**Affiliations:** 1 Escola Superior de Saúde, Polytechnic Institute of Santarém, 2001-904 Santarém, Portugal; alcinda.reis@essaude.ipsantarem.pt (A.R.); celeste.godinho@essaude.ipsantarem.pt (C.G.); ana.madeira@essaude.ipsantarem.pt (A.C.S.M.); 2 RISE-Health, School of Health, Santarém Polytechnic University, 2005-075 Santarém, Portugal

**Keywords:** informal caregiver, health literacy, primary healthcare, nursing, mixed methods

## Abstract

**Highlights:**

**What are the main findings?**

- In this single-site exploratory sample, 22 of 30 informal caregivers (73.3%; 95% CI 55.6–85.8) had limited general health literacy, and 28 of 30 (93.3%; 95% CI 78.7–98.2) had limited health literacy in the health-promotion domain.
- The QUAL phase added interpretive depth by showing how informal caregivers from the highest recorded caregiving-intensity category described clear, personalised, home-based nursing communication, decision support and resource guidance as helpful, while also reporting unmet needs related to time, explanation and proactive teaching.

**What are the implications of the main findings?**

- The findings support nursing attention to health-literacy-responsive communication, caregiver education, desired checking of understanding, decision support and informal caregivers’ own health and self-care.
- Because no intervention effect or participant-level relationship was measured, practice recommendations are presented as literature-informed and caregiver-informed implications requiring future evaluation.

**Abstract:**

Background/Objectives: Informal caregivers support home care while having health, information and self-care needs. This study assessed general and domain-specific health literacy among informal caregivers registered with a Portuguese Family Health Unit and explored, with QUAL interpretive priority for the nursing-practice objective, perceptions of nursing practices supportive of health literacy. Methods: A sequential mixed-methods design (quan → QUAL) was used. The quantitative phase included 30 informal caregivers who completed a sociodemographic questionnaire and the Portuguese European Health Literacy Survey (HLS-EU-PT). The QUAL phase included five informal caregivers selected from the highest recorded caregiving-intensity category and interviewed at home. Quantitative data were analysed descriptively; 95% confidence intervals were calculated for principal means and proportions. Qualitative data were analysed using Bardin’s content analysis. Integration occurred through connection and aggregate interpretation; individual HLS-EU-PT scores were not linked to interviews. Results: Informal caregivers were mostly women (83.3%), older than 55 years (80.0%) and cohabiting with the dependent person (83.3%). The mean general HLS-EU-PT score was 26.64 ± 8.40 (95% CI 23.50–29.78); 22/30 informal caregivers had limited health literacy (73.3%; 95% CI 55.6–85.8). Health promotion was the lowest-scoring domain (25.34 ± 8.31; 95% CI 22.24–28.44), with 28/30 informal caregivers showing limited health literacy (93.3%; 95% CI 78.7–98.2). QUAL findings highlighted personalised information, checking understanding, caregiver experience, decision support and resource guidance. Conclusions: Limited health literacy was frequent. The QUAL phase deepened understanding of perceived nursing support, but findings indicate perceived needs rather than effects.

## 1. Introduction

Population ageing, the increasing prevalence of chronic diseases, and rising functional dependence have expanded the need for continuing care at home. A substantial proportion of this care is provided by family members or other close persons, often without specific training and with limited formal support. Informal caregivers undertake tasks related to activities of daily living, therapeutic regimen management, monitoring of signs and symptoms, prevention of complications and coordination with health and social services. Such responsibilities require knowledge, practical skills, decision-making capacity and timely access to information that is understandable and applicable to the home context [1].

Informal caregivers should not be regarded only as a resource for the dependent person. They are also people with their own health needs, exposed to physical and emotional strain and potential limitations in self-care. High-intensity caregiving may reduce time and energy for prevention and health promotion, especially when care is provided for many hours per day and when the caregiver cohabits with the dependent person. In this context, health literacy is relevant not only for care tasks but also for the caregiver’s own health, help-seeking capacity and ability to navigate health and social services.

Health literacy is commonly understood as the knowledge, motivation and competencies required to access, understand, appraise and apply health information when making decisions related to healthcare, disease prevention and health promotion [2]. Limited health literacy has been associated with poorer use of preventive information, higher demand for urgent services and lower capacity to act on health information [3,4]. Among informal caregivers, health literacy can influence communication with professionals, treatment management, recognition of warning signs, navigation of resources and the balance between caring for another person and self-care [5,6,7].

Health literacy is also relational and organisational. It is shaped by how health services communicate, organise information, facilitate access, check understanding and support decision-making. This dimension is particularly relevant in primary healthcare, where nurses often maintain close and continuing contact with families, including through home nursing consultations. Portuguese health-literacy strategies emphasise the identification of vulnerable groups, the adaptation of communication and the development of tools that help professionals make information usable and actionable [8,9].

Previous studies have described health literacy in general populations and among informal caregivers. However, less is known about how domain-specific health-literacy results coexist with informal caregivers’ concrete experiences of communication, learning, decision-making and perceived nursing support in primary care. The present study was grounded in a master’s dissertation that defined not only the assessment of informal caregivers’ health literacy, but also the identification of nursing care practices perceived as promoting informal caregivers’ health literacy. This dual objective justified a sequential mixed-methods design in which the quan phase provided local descriptive measurement and the QUAL phase was given interpretive priority for understanding informal caregivers’ experiences and perceived support needs.

The overall aim was to assess general and domain-specific health literacy among informal caregivers registered with a Family Health Unit and to explore informal caregivers’ perceptions of nursing practices perceived as supportive of health literacy. The research questions were as follows: (1) What are informal caregivers’ levels of general and domain-specific health literacy? (2) Which nursing practices are perceived as facilitating understanding, decision-making and care management? (3) How do the QUAL findings complement the quantitative health-literacy results at aggregate level?

## 2. Materials and Methods

### 2.1. Study Design and Setting

A sequential mixed-methods design was adopted, represented as quan → QUAL. The quantitative phase provided a local descriptive assessment of informal caregivers’ general and domain-specific health literacy. The subsequent QUAL phase, conducted with informal caregivers drawn from the quantitative sample, explored informal caregivers’ perceptions of nursing practices, learning experiences, autonomy and decision-making. The qualitative component had interpretive priority for the study’s nursing-practice objective and for translating the quantitative profile into practice-relevant understanding.

This choice does not mean that the QUAL phase fully explained individual HLS-EU-PT scores. Qualitative participants were selected from the highest recorded caregiving-intensity category rather than by health-literacy profile, and individual questionnaire scores were not linked to interview identifiers. Accordingly, the study is sequential and mixed, but not a fully explanatory participant-level design. Integration occurred through connection between phases, aggregate interpretation and integrated discussion, following mixed-methods principles of connection and reporting [10].

The study was conducted in a Portuguese Family Health Unit selected because of the researcher’s access to the setting and the dependency profile of its registered population. This local context supports the relevance of the study but limits transferability.

### 2.2. Informal Caregivers, Sampling and Recruitment

The target population comprised informal caregivers of dependent persons registered with the Family Health Unit. The initial sampling frame was obtained from the MIM@UF monitoring system and included users with the “Dependent Persons” programme active and with home nursing consultations recorded during 2022. The source list was generated from dependent persons and then verified to identify the corresponding informal caregiver. Study participation and analysis were defined at caregiver level; when a caregiver was associated with more than one dependent person, that person was counted once as a study participant.

Of 54 dependent persons initially identified, five were excluded because they were institutionalised and had formal caregivers. Among the remaining 49 dependent persons with an informal caregiver, eight informal caregivers no longer met eligibility criteria at the time of the study: four informal caregivers had died, two had written-communication deficits and two cared for users registered in another unit at the study date. The final eligible population comprised 41 informal caregivers. Inclusion criteria were as follows: being an informal caregiver, being aged 18 years or older, voluntarily agreeing to participate and being able to read and write in Portuguese. Exclusion criteria were failure to meet these criteria or oral/written communication deficits preventing participation.

The quantitative sample consisted of 30 informal caregivers. No formal power calculation was performed because the study was local, exploratory and descriptive. Thirty informal caregivers were selected as a feasible sample within the authorised data-collection period and field resources, while still representing 73.2% of the eligible population. Simple random sampling was performed manually: each eligible caregiver was assigned a number, the 41 numbers were placed in a bag, and numbers were drawn until 30 informal caregivers were selected. All selected informal caregivers were contacted by telephone by the researcher and agreed to participate.

For the QUAL phase, criterion-based purposive sampling was used. The quantitative questionnaire recorded daily caregiving time in categorical bands. The five interviewees were therefore selected from participants in the highest recorded caregiving-intensity category (12–24 h/day) who consented to be interviewed and were available for a home interview. The selection criterion was grounded in the study’s purpose of exploring informal caregivers with substantial day-to-day exposure to care demands and was the closest operational approximation to the concept of principal caregiver. Exact continuous caregiving hours were not recorded or used to rank participants within this category; selection within the category was therefore based on consent and availability during the authorised data-collection period. This option may have privileged experiences associated with high caregiving intensity and limited diversity of perspectives. Formal saturation is not claimed.

Table 1 summarises the flow from the initial sampling frame to the quantitative and QUAL samples.

### 2.3. Instruments and Data Collection

Quantitative data were collected in March 2024 using a sociodemographic questionnaire and the Portuguese version of the European Health Literacy Survey (HLS-EU-PT), validated for the Portuguese population [4]. The HLS-EU-PT comprises 47 items distributed across three domains: healthcare, disease prevention and health promotion. Items are answered on a four-point scale from ‘very difficult’ to ‘very easy’. The sociodemographic questionnaire included gender, age, schooling, marital status, employment status, relationship with the dependent person, cohabitation, number of persons cared for, duration of caregiving, daily caregiving time and age of the dependent person.

HLS-EU-PT indices were standardised on a 0–50 scale using I = [(X − 1)/3] × 50, where X is the respondent’s mean item score for the relevant index. Levels were classified as inadequate (0–25), problematic (>25–33), sufficient (>33–42) and excellent (>42–50). Inadequate and problematic categories were aggregated as limited health literacy [3]. No missing data were identified in the questionnaires included in the analysis. The Portuguese validation study reported Cronbach’s alpha of 0.96 for the 47-item scale [4]. Internal consistency was not recalculated for the present sample because the original study report did not include item-level reliability analysis for this sample and the revision did not have a re-analysable item-level dataset.

QUAL data were collected through semi-structured interviews guided by 11 questions aligned with the study objectives and the health-literacy framework. The guide explored the initial caregiving phase, learning process, sources of information, differences between recommended and feasible care, information about prevention and complications, access to updated information, clarification of doubts, decision-making, desired nursing support, and what should be taught to new informal caregivers. The full guide is provided as Supplementary File S1. The guide was piloted with two informal caregivers with similar characteristics to the inclusion criteria; no comprehension difficulties were identified and no changes were made. Pilot interviews were not included in the final analysis. The five study interviews took place at informal caregivers’ homes in April and May 2024, at times considered appropriate by informal caregivers. Interviews lasted approximately 25 min on average, were audio-recorded, and were transcribed verbatim by the principal investigator.

The principal investigator was a nurse and master’s student in Community Nursing and was familiar with the institutional setting. During interviews, she used open questions, clarification prompts and active listening, seeking to avoid leading formulations. Informal caregivers were informed about voluntariness, confidentiality, pseudonymisation and the right to withdraw, and were asked not to mention identifying names. Identifiers mentioned in the recordings were removed during transcription.

### 2.4. Data Analysis and Integration

Quantitative data were processed using IBM SPSS Statistics for Windows, version 22.0. Descriptive statistics included absolute and relative frequencies, means, standard deviations, minima and maxima. Percentages were recalculated using *n* = 30 and a consistent one-decimal rounding convention. To quantify precision for the principal descriptive estimates, t-based 95% confidence intervals were calculated for means from *n*, mean and SD, and Wilson 95% confidence intervals were calculated for key proportions from the numerator and denominator. Medians, interquartile ranges and current-sample Cronbach’s alpha were not calculated because the available archived material did not include a re-analysable participant-level score distribution or item-level HLS-EU-PT matrix.

Qualitative data were analysed according to Bardin’s content analysis approach [11]. In this study, the recording unit, used as the unit of analysis, corresponded to a segment of discourse with autonomous meaning in relation to the study objectives. The analysis followed the stages of pre-analysis, exploration of the material, coding, categorisation and interpretation. The process was hybrid: the health-literacy operations of accessing, understanding, appraising and applying information acted as sensitising concepts, while categories and subcategories were refined inductively from informal caregivers’ accounts. The first author conducted transcription, familiarisation and initial coding. The supervisory team reviewed the coding matrix, category definitions and selected excerpts; this is described as matrix review and interpretive discussion, not as independent full double coding.

Mixed-methods integration was conducted at aggregate level. The QUAL phase was used to deepen interpretation of the quantitative profile by identifying the communication, learning, decision-making and support issues described by informal caregivers from the highest recorded caregiving-intensity category. Because individual HLS-EU-PT scores were not linked to interview identifiers, the study does not claim to explain why specific informal caregivers had inadequate, problematic or sufficient health-literacy scores.

### 2.5. Ethical Considerations

The study complied with the principles of the Declaration of Helsinki and applicable confidentiality and data-protection requirements. Authorisation to use the HLS-EU-PT was obtained, as were a favourable opinion from the Health Ethics Committee of the competent Regional Health Administration, approval from the executive body of the Health Centre Group, and authorisation from the coordinator of the Family Health Unit. The Ethics Committee issued favourable opinion no. 960/CES/2024.

All informal caregivers received information about the study objectives, voluntary participation, the right to withdraw, confidentiality, pseudonymisation and data processing, and provided informed consent. Data were pseudonymised using alphanumeric codes. According to the approved protocol reported in the dissertation, audio recordings were destroyed after transcription and analysis. Paper questionnaires and other research documents were stored securely by the principal investigator during the study period and scheduled for destruction after study completion. Only aggregate quantitative outputs and de-identified qualitative materials were used for this revision.

## 3. Results

### 3.1. Quantitative Participant Characteristics

The quantitative sample included 30 informal caregivers. Table 2 presents the complete distribution available in the dissertation source. The sample was predominantly female, older than 55 years, frequently cohabiting with the dependent person and characterised by high daily caregiving intensity.

### 3.2. General and Domain-Specific Health Literacy

The mean overall HLS-EU-PT score was 26.64 ± 8.40 (95% CI 23.50–29.78; range 8.51–39.36), corresponding to problematic health literacy. At category level, 11 informal caregivers (36.7%) had inadequate health literacy, 11 (36.7%) had problematic health literacy, eight (26.7%) had sufficient health literacy and none had excellent health literacy. Thus, 22 of 30 informal caregivers had limited general health literacy (73.3%; 95% CI 55.6–85.8).

Health promotion was the lowest-scoring domain and the domain with the highest proportion of limited health literacy. Its mean score was 25.34 ± 8.31 (95% CI 22.24–28.44; range 4.17–38.54). According to the stated thresholds, this mean falls within the problematic category (>25–33), not the inadequate category. In this domain, 28 of 30 informal caregivers (93.3%; 95% CI 78.7–98.2) had limited health literacy. Healthcare and disease prevention also had mean scores in the problematic range.

Table 3 summarises the overall and domain-specific HLS-EU-PT scores, classifications and category counts.

### 3.3. QUAL Participant Context

The QUAL component included five informal caregivers selected from the highest recorded caregiving-intensity category. The available qualitative material did not include participant-level HLS-EU-PT scores linked to interview identifiers. Table 4 therefore presents only the interview-context characteristics that can be derived from the qualitative accounts without reconstructing unverified individual profiles.

### 3.4. QUAL Findings

The QUAL component included five informal caregivers selected from the highest recorded caregiving-intensity category. The analysis identified two overarching themes: facilitating learning environments and nursing practices perceived as supportive of health literacy.

Divergent or less favourable accounts were treated as a cross-cutting analytic qualifier, not as a third theme. The analytic narrative below explains how categories related to each other and includes examples from all five interviewees.

#### 3.4.1. Facilitating Learning Environments

The first theme concerns the conditions that made learning possible in the home-care context. Informal caregivers valued information that was clear, specific and adapted to the dependent person’s situation. E4 stated that nurses “explained everything that was happening”, while E5 valued information that was “more directed to our individual situation”. These accounts link clarity to personalisation: information was perceived as more useful when it referred to the actual disease trajectory, symptoms, routines and physical conditions observed at home.

The interviews also suggested that explanation alone was not always sufficient. E2 described reluctance to ask questions because she did not want to “bother” professionals and argued that professionals should ask informal caregivers what they had understood: “I have been talking to you; what did you understand?” This quotation supports the category as a caregiver-expressed need for checking understanding, not as evidence that a formal teach-back intervention was implemented or evaluated.

Autonomy-supportive procedures were closely linked to relational availability. E1 described having “a shoulder” that helped her day-to-day coping, while E4 described accumulating learning through repeated contacts with different nurses. E3, however, emphasised that she had “learned a little alone” when suddenly caring for both parents. These accounts show convergence around the value of support, but variation in how much proactive teaching informal caregivers perceived receiving.

#### 3.4.2. Nursing Practices Perceived as Supportive of Health Literacy

The second theme concerns practices that informal caregivers perceived as supportive when they needed to appraise information and apply it to daily care decisions. Decision support was particularly evident in E5’s account of catheterisation and hospital referral decisions, described as decisions discussed with professionals and experienced as relieving. E4 also associated accumulated learning from nurses with being better able to make decisions over time.

Resource navigation appeared as another component of perceived support. E5 reported receiving contacts for support groups, information about technical aids and indications about reliable sources. E4, by contrast, emphasised the difficulty of finding “official” information and warned that online information is often not trustworthy. These accounts converge on the need for guidance, but differ in whether informal caregivers experienced resource navigation as readily available or difficult to obtain.

Informal caregivers also highlighted the need to adapt recommendations to the domestic context. E3 stated that “if everything is explained, I can do it”, whereas E5 noted that “what works for one person does not work for another”. This variation shows that the perceived usefulness of nursing guidance depended on whether information could be translated into feasible actions within the caregiver’s actual circumstances.

#### 3.4.3. Cross-Cutting Less Favourable Accounts

Less favourable accounts qualified the generally positive view of nursing support. E1 reported encounters that were “a little too rushed” and “did not help”. E2 similarly argued that professionals should “talk more” and “teach the person how we should do it”. E4 stated that nurses provide necessary information but that “small nuances” may not be offered unless the caregiver asks. These excerpts caution against interpreting the results as evidence that existing support was consistently sufficient. The QUAL findings identify experiences, preferences and unmet needs, not intervention effectiveness.

Table 5 summarises the qualitative coding structure and evidentiary support.

### 3.5. Aggregate Mixed-Methods Integration

Table 6 presents the aggregate mixed-methods joint display, distinguishing direct quantitative findings, direct QUAL findings, cautious meta-inferences and implications. Integration is aggregate and interpretive. No individual-level relationship was examined between HLS-EU-PT scores, caregiving intensity and interview accounts.

## 4. Discussion

This study found a high frequency of limited perceived health literacy among informal caregivers in one Family Health Unit, with health promotion emerging as the lowest-scoring domain and the domain with the highest proportion of limited health literacy. The quan phase showed that 22 of 30 informal caregivers had limited general health literacy and 28 of 30 had limited health literacy in health promotion. The QUAL phase added interpretive depth by showing how informal caregivers from the highest recorded caregiving-intensity category described communication, learning, decision-making and support needs in the context of home care.

The observed proportion of limited health literacy appears higher than values reported in Portuguese population studies and in some caregiver studies [4,6,7]. However, the comparison must be cautious. The present sample was small, local and drawn from informal caregivers of dependent persons associated with home nursing care. It should therefore be interpreted as a signal of local vulnerability rather than an epidemiological estimate. The study also excluded informal caregivers who could not read or write Portuguese or had communication deficits, a criterion that may have led to underestimation rather than overestimation of vulnerability.

The low score in health promotion is clinically relevant because many informal caregivers’ daily concerns are centred on immediate care tasks. The QUAL accounts frequently addressed symptoms, feeding, catheterisation, hygiene, decision-making, technical aids and access to services. Informal caregivers’ own health and self-care appeared less central in the interviews. This does not prove that caregiving intensity causes poorer health-promotion literacy; such a relationship was not measured. It suggests, rather, that health-promotion information may be less visible or less actionable when the caregiver is absorbed by urgent and continuous care demands.

The QUAL findings also clarify that nursing support was not experienced only as information transmission. Informal caregivers valued explanations adapted to the dependent person, teaching in the home context, availability to clarify doubts, support in deciding when to seek care and guidance towards reliable information and social resources. These findings align with the broader view that health literacy depends partly on the responsiveness of services and professionals, not only on the individual’s abilities [8,9,12,13,14,15].

Nevertheless, the study does not demonstrate that specific nursing practices improved health literacy. It refers to practices perceived as supportive of health literacy, experiences of nursing support and perceived support needs. This distinction is important because the study did not include an intervention, a pre-post evaluation or a participant-level mixed-methods analysis linking HLS-EU-PT scores to interview accounts.

The less favourable accounts are important. Some informal caregivers described professionals as rushed, insufficiently explanatory or dependent on the caregiver asking questions. These accounts suggest that health-literacy-responsive support should not rely only on caregiver initiative. Informal caregivers with lower confidence, lower schooling, greater emotional burden or communication difficulties may be less able to ask for clarification. This is especially relevant because some of the most vulnerable informal caregivers could not be included under the study protocol.

The qualitative phase was not an accessory illustration of the survey results; it addressed a central objective of the dissertation: identifying nursing practices perceived by informal caregivers as supporting health literacy. The QUAL phase had interpretive priority for understanding how informal caregivers made sense of information, support and decision-making in their daily caregiving context. At the same time, this priority is limited by the small number of interviews, the selection of participants from the highest caregiving-intensity category, and the absence of participant-level linkage with HLS-EU-PT scores.

## 5. Implications for Practice

The findings suggest areas for nursing attention in Family Health Units. First, informal caregivers should be assessed as people with their own health, self-care and information needs, not only as persons who provide care to someone dependent. This is particularly important when informal caregivers provide 12–24 h of daily care and cohabit with the dependent person.

Second, nursing communication should be designed to be understandable and usable. The present interviews support the importance of clear and personalised explanation, home-based teaching, opportunities to ask questions and checking what the caregiver understood. Recommendations such as plain language, teach-back, visual materials and written action plans are supported mainly by the wider health-literacy literature [9,12,13,14,15] and by informal caregivers’ expressed needs, but their effectiveness was not tested in this study.

Third, resource navigation should be integrated into caregiver support. Informal caregivers described the usefulness of guidance towards reliable information, technical aids, day-care support, informal caregiver status and support groups. This is consistent with caregiver support and empowerment-oriented interventions and reviews [16,17,18,19]. Future research should test whether structured navigation tools improve informal caregivers’ knowledge, self-efficacy, self-care, care safety or service use.

Future studies should use multicentre samples, retain participant-level linkage between quantitative and qualitative data, include informal caregivers with different health-literacy profiles, and adopt accessible data-collection procedures for people with communication or literacy difficulties. Intervention studies are needed before broader claims can be made about effectiveness.

## 6. Limitations

This study has several limitations. It was conducted in a single Family Health Unit, with a small quantitative sample and five interviews, which limits transferability. The sampling frame was constructed from patients with home nursing consultations in 2022, whereas data were collected in 2024; informal caregivers who entered the programme after 2022 were not included in the predefined frame.

The sample size was feasibility-based, and no formal statistical power calculation was performed. Confidence intervals were added for principal descriptive estimates because they could be derived from aggregate counts, means and standard deviations. However, medians, interquartile ranges and current-sample Cronbach’s alpha could not be calculated because the available material did not include a re-analysable participant-level score distribution or item-level HLS-EU-PT matrix.

The requirement to be able to read and write in Portuguese, together with the exclusion of informal caregivers with communication deficits, may have excluded individuals at higher risk of limited health literacy. The HLS-EU-PT assesses perceived difficulty rather than objective health-literacy performance.

The QUAL phase involved only five informal caregivers selected from the highest recorded caregiving-intensity category. This option allowed depth within a highly exposed subgroup but restricted the diversity of perspectives. Formal saturation is not claimed. The available documentation does not report systematic field notes, transcript return, or independent full double coding. The researcher’s familiarity with the setting and informal caregivers’ relationship with the Family Health Unit may have contributed to socially desirable responses. Finally, individual HLS-EU-PT scores were not linked to interview accounts, preventing mixed-methods integration at participant level.

## 7. Conclusions

In this local exploratory sequential mixed-methods study, limited perceived health literacy was frequent among informal caregivers, particularly in the health-promotion domain. The QUAL phase, prioritised for interpretive depth in relation to the nursing-practice objective, showed that informal caregivers valued clear and personalised communication, home-based education, opportunities to clarify doubts, decision support and guidance towards resources, while also reporting unmet needs related to time, proactive explanation and checking understanding.

The findings support closer attention to informal caregivers’ own health-literacy needs and to the communicative accessibility of nursing support in primary care. However, the study identifies perceived support needs and experiences, not the effectiveness of nursing interventions. Its conclusions are limited by the local setting, small sample, categorical selection of qualitative participants, exclusion criteria, short qualitative component, and absence of participant-level quantitative–qualitative integration. Larger multicentre and intervention studies are needed before broader clinical recommendations can be made.

## Figures and Tables

**Table 1:** Participant flow from sampling frame to analysed samples.

Flow Element	*n*
Initial list of dependent persons with programme active and home nursing consultations in 2022	54
Excluded: institutionalised persons with formal caregivers	5
Dependent persons with an informal caregiver	49
Excluded before recruitment: caregiver died (*n* = 4), written-communication deficit (*n* = 2), user registered in another unit at study date (*n* = 2)	8
Eligible informal caregivers	41
Randomly selected, contacted, consenting and analysed in the quantitative phase	30
Selected from highest caregiving-intensity category and interviewed in QUAL phase	5

**Table 2:** Sociodemographic and caregiving characteristics of quantitative informal caregivers (*n* = 30).

Variable	Category	*n*	%
Age group	26–35 years	1	3.3
Age group	36–45 years	0	0.0
Age group	46–55 years	5	16.7
Age group	56–65 years	10	33.3
Age group	>65 years	14	46.7
Gender	Male	5	16.7
Gender	Female	25	83.3
Marital status	Married/cohabiting	18	60.0
Marital status	Single	3	10.0
Marital status	Divorced/separated	4	13.3
Marital status	Widowed	5	16.7
Education	1st cycle (up to 4th grade)	9	30.0
Education	2nd cycle (5th–6th grade)	4	13.3
Education	3rd cycle (7th–9th grade)	7	23.3
Education	Secondary education	4	13.3
Education	Higher education	6	20.0
Employment status	Paid employment/profit	12	40.0
Employment status	Unemployed	1	3.3
Employment status	Retired	16	53.3
Employment status	Other situation	1	3.3
Relationship to dependent person	Spouse/partner	13	43.3
Relationship to dependent person	Child	13	43.3
Relationship to dependent person	Sibling	1	3.3
Relationship to dependent person	No kinship	1	3.3
Relationship to dependent person	Parent	2	6.7
Number of persons cared for	1 person	26	86.7
Number of persons cared for	2 persons	3	10.0
Number of persons cared for	3 persons	1	3.3
Duration of caregiving	0–2 years	5	16.7
Duration of caregiving	3–5 years	17	56.7
Duration of caregiving	6–10 years	4	13.3
Duration of caregiving	>10 years	4	13.3
Daily caregiving time	0–2 h/day	1	3.3
Daily caregiving time	3–5 h/day	4	13.3
Daily caregiving time	6–8 h/day	3	10.0
Daily caregiving time	9–11 h/day	1	3.3
Daily caregiving time	12–24 h/day	21	70.0
Dependent person age	0–25 years	2	6.7
Dependent person age	26–65 years	2	6.7
Dependent person age	66–80 years	6	20.0
Dependent person age	>81 years	20	66.7
Cohabitation with dependent person	Yes	25	83.3
Cohabitation with dependent person	No	5	16.7

**Table 3:** HLS-EU-PT results: means, levels and category counts (*n* = 30).

Index/Domain	Mean ± SD; 95% CI; Range	Mean Level	Inadequate *n* (%)	Problematic *n* (%)	Sufficient *n* (%)	Excellent *n* (%)	Limited *n* (%)
Overall health literacy	26.64 ± 8.40; 95% CI 23.50–29.78; range 8.51–39.36	Problematic	11 (36.7)	11 (36.7)	8 (26.7)	0 (0.0)	22 (73.3; 95% CI 55.6–85.8)
Healthcare	26.73 ± 8.47; 95% CI 23.57–29.89; range 10.42–39.58	Problematic	13 (43.3)	10 (33.3)	7 (23.3)	0 (0.0)	23 (76.7; 95% CI 59.1–88.2)
Disease prevention	27.92 ± 9.48; 95% CI 24.38–31.46; range 10.00–44.44	Problematic	12 (40.0)	10 (33.3)	7 (23.3)	1 (3.3)	22 (73.3; 95% CI 55.6–85.8)
Health promotion	25.34 ± 8.31; 95% CI 22.24–28.44; range 4.17–38.54	Problematic	10 (33.3)	18 (60.0)	2 (6.7)	0 (0.0)	28 (93.3; 95% CI 78.7–98.2)

Note: Limited health literacy = inadequate + problematic. Percentages for limited health literacy were calculated directly from the count and denominator, not by summing rounded category percentages. Confidence intervals for means are t-based 95% CIs calculated from *n*, mean and SD. Confidence intervals for proportions are Wilson 95% CIs calculated from numerator and denominator.

**Table 4:** Available context of QUAL interviewees.

Code	Caregiving Context from Interview Account	Selection Basis	Interview Setting/Language	Analytic Contribution
E1	Spouse caregiver; previous family caregiving experience	Highest recorded caregiving-intensity category	Home; Portuguese; approx. 25 min average across interviews	Described both valued support and rushed professional contacts
E2	Spouse caregiver of an older husband	Highest recorded caregiving-intensity category	Home; Portuguese; approx. 25 min average across interviews	Emphasised shame/reluctance to ask questions and need for proactive explanation
E3	Adult child caregiver caring for father and mother	Highest recorded caregiving-intensity category	Home; Portuguese; approx. 25 min average across interviews	Stressed learning “alone” and needing practical home training
E4	Male adult child caregiver caring for mother	Highest recorded caregiving-intensity category	Home; Portuguese; approx. 25 min average across interviews	Valued accumulated learning from nurses but highlighted difficulty obtaining official information
E5	Adult child caregiver caring for father	Highest recorded caregiving-intensity category	Home; Portuguese; approx. 25 min average across interviews	Described personalised guidance, resource navigation and shared decisions

Note: Demographic details and individual HLS-EU-PT scores were not linked to interview codes in the available manuscript dataset; the table avoids adding unverified participant-level data.

**Table 5:** QUAL coding structure and evidentiary support.

Theme/Analytic Status	Category	Subcategory Focus	Coding Basis	Illustrative Quotations
Facilitating learning environments	Perceived communication techniques	Clear/personalised information; desired checking of understanding	Deductive sensitising concept: understanding information; inductive refinement from accounts of explanation and adaptation	“They explained everything that was happening” (E4); “the information they gave us was more directed to our individual situation” (E5); “What did you understand?” as desired checking (E2).
Facilitating learning environments	Autonomy-supportive procedures	Valuing caregiver experience; relational support; support networks	Inductive refinement from accounts of being listened to, supported and connected	“I had a shoulder there… it helped me improve day to day” (E1); “I learned a little alone” (E3); “they provided contacts for some support groups” (E5).
Nursing practices perceived as supportive of health literacy	Decision support	Confidence/security; shared decisions; problem solving	Deductive sensitising concepts: appraising and applying information; inductive accounts of uncertainty and decisions	“When it was the catheterisation moment, we decided together” (E5); accumulated learning helped decision-making (E4).
Nursing practices perceived as supportive of health literacy	Guidance towards resources	Health/social resources; reliable information; visual/technological materials	Inductive accounts of navigating services, official information and community support	“They told me where I could seek more information” (E5); difficulty finding official information and unreliable internet sources (E4).
Nursing practices perceived as supportive of health literacy	Behavioural adaptation	Implementation of recommendations; integration of alternatives	Deductive sensitising concept: applying information; inductive accounts of adapting care to context	“If everything is explained, I can do it” (E3); “what works for one person does not work for another” (E5).
Cross-cutting analytic qualifier	Perceived insufficient support	Rushed professionals; insufficient explanation; desire for proactive teaching	Inductive category retained to avoid overly favourable interpretation	“A little too rushed for my liking and it did not help me” (E1); “They should talk more… teach the person how we should do it” (E2).

**Table 6:** Aggregate mixed-methods joint display.

Direct Quantitative Finding	Direct QUAL Finding	Cautious Meta-Inference	Practice/Research Implication
22/30 informal caregivers had limited general health literacy (73.3%; 95% CI 55.6–85.8).	Informal caregivers valued clear explanations, personalisation and opportunities to ask questions.	Limited health literacy was frequent, and informal caregivers from the highest recorded caregiving-intensity category described communication conditions that made information more usable.	Use plain language and check comprehension as literature-informed and caregiver-informed strategies; the study did not test effectiveness.
28/30 informal caregivers had limited health literacy in health promotion (93.3%; 95% CI 78.7–98.2).	Interviews centred on daily care tasks, symptoms, decision-making and resources; caregiver self-care was less visible.	The health-promotion vulnerability may coexist with the dominance of immediate care demands, but this relationship was not directly measured.	Include caregiver health and self-care in nursing assessment and future intervention studies.
21/30 informal caregivers provided 12–24 h/day of care.	QUAL interviewees were selected from the highest recorded caregiving-intensity category.	Convergence between the highest recorded caregiving-intensity category and accounts of home support is partly built into sampling and must be interpreted cautiously.	Future studies should retain participant-level linkage and sample interviewees by health-literacy profile and caregiving intensity.
Healthcare and disease-prevention scores were problematic.	Informal caregivers described needing help to decide when to seek services, manage treatment routines and identify complications.	Professional support may help informal caregivers appraise and apply information, but compensatory effects were not tested.	Develop and evaluate decision-support tools, written action plans and resource-navigation guidance.

## Data Availability

Aggregate quantitative data and de-identified qualitative excerpts are presented in this manuscript and in the source dissertation. Individual participant data are not publicly available because of confidentiality and data-protection requirements. The revision was based on the dissertation, aggregate quantitative outputs and de-identified qualitative material; a re-analysable participant-level HLS-EU-PT item matrix was not available at the revision stage. Requests for access to non-public research materials should be directed to the corresponding author and are subject to institutional and ethical approvals where applicable.

## Supplementary Materials

The following supporting information can be downloaded at: https://www.mdpi.com/article/10.3390/healthcare14182987/s1, Supplementary File S1: Semi-structured interview guide.
