# Supplementary material for: Informal Caregivers’ Health Literacy and Nursing Practices Perceived as Supportive of Health Literacy in Primary Care: A Sequential Mixed-Methods Study
Source: Healthcare (Basel). 2026 Sep 12;14(18):2987. doi: 10.3390/healthcare14182987 (PMC13606594; doi:10.3390/healthcare14182987)
Supplement: Supplementary file 1 [file healthcare-14-02987-s001.zip › healthcare-4489256-supplementary.pdf]

Supplementary File S1

Semi-structured interview guide

Topic: Health literacy of informal caregivers

Objective: To identify informal caregivers’ perceptions regarding nursing support and practices perceived as supportive of health literacy.

1. How was the initial phase of your role as caregiver? What difficulties did you experience? How did you overcome those initial difficulties?
2. Can you talk a little about your learning process? How did you learn, with whom, and can you give examples of things you learned?
3. Who do you contact when you have doubts?
4. Are there differences between what you are advised to do and what you can actually do in reality? Can you give examples?
5. How do nurses inform you about where to obtain information on preventing diseases or complications in the dependent person you care for?
6. Do nurses help improve your access to up-to-date health information and help improve your day-to-day care?
7. How do nurses clarify your doubts about health information, contributing to improve your daily caregiving?
8. How do nurses clarify the information or doubts you need in order to make better decisions about the health of the person you care for?
9. How do nurses help you improve your decision-making capacity regarding health issues affecting the person you care for?
10. What do you consider important to teach someone who is starting to provide care to a dependent person?
11. Would you like to add any information that has not been asked?

Note: The original interview guide was written in Portuguese. This version is an English translation prepared for the manuscript resubmission.
